# Short-term outcomes of cadaveric lung transplantation in ventilator-dependent patients

**DOI:** 10.1186/cc7989

**Published:** 2009-08-06

**Authors:** Hsao-Hsun Hsu, Jin-Shing Chen, Wen-Je Ko, Shu-Chien Huang, Shuenn-Wen Kuo, Pei-Ming Huang, Nai-Hsin Chi, Chin-Chih Chang, Robert J Chen, Yung-Chie Lee

**Affiliations:** 1 Department of Surgery, National Taiwan University Hospital and National Taiwan University College of Medicine, No. 7, Chung-Shan South Road, Taipei City, 100, Taiwan; 2 Department of Traumatology, National Taiwan University Hospital and National Taiwan University College of Medicine, No. 7, Chung-Shan South Road, Taipei City, 100, Taiwan; 3 Institute of Epidemiology, College of Medicine, National Taiwan University, No. 1, Section 1, Ren-Ai Road, Taipei City, 100, Taiwan

## Abstract

**Introduction:**

Survival after cadaveric lung transplantation (LTx) in respiratory failure recipients who were already dependent on ventilation support prior to transplantation is poor, with a relatively high rate of surgical mortality and morbidity. In this study, we sought to describe the short-term outcomes of bilateral sequential LTx (BSLTx) under extracorporeal membrane oxygenation (ECMO) support in a consecutive series of preoperative respiratory failure patients.

**Methods:**

Between July 2006 and July 2008, we performed BSLTx under venoarterious (VA) ECMO support in 10 respiratory failure patients with various lung diseases. Prior to transplantation, 6 patients depended on invasive mechanical ventilation support and the others (40%) needed noninvasive positive pressure ventilation to maintain adequate gas exchange. Their mean age was 40.9 years and the mean observation period was 16.4 months.

**Results:**

Except for 1 ECMO circuit that had been set up in the intensive care unit for pulmonary crisis 5 days prior to transplantation, most ECMO (90%) circuits were set up in the operating theater prior to pneumonectomy of native lung during transplantation. Patients were successfully weaned off ECMO circuits immediately after transplantation in 8 cases, and within 1 day (1/10 patients) and after 9 days (1/10 patients) due to severe reperfusion lung edema following transplantation. The mean duration of ECMO support in those successfully weaned off in the operating theater (n = 8) was 7.8 hours. The average duration of intensive care unit stay (n = 10) was 43.1 days (range, 35 to 162 days) and hospital stay (n = 10) was 70 days (range, 20 to 86 days). Although 4 patients (40%) had different degrees of complicated postoperative courses unrelated to ECMO, all patients were discharged home postoperatively. The mean forced vital capacity and the forced expiratory volume in 1 second both increased significantly postoperatively. The cumulative survival rates at 3 months and at 12 months post-transplantation were 100% and 90%.

**Conclusions:**

Although BSLTx in this critical population has varied surgical complications and prolonged length of postoperative ICU and hospital stays, all the patients observed in this study could tolerate the transplant procedures under VA ECMO support with promising pulmonary function and satisfactory short-term outcome.

## Introduction

Lung transplantation (LTx) has been performed internationally as a viable, life-saving intervention for a variety of end-stage lung diseases. However, ventilator dependency while on the waiting list is still considered to be a relative or absolute contraindication to LTx by most centers, because of concerns regarding the possible risk of post-transplant pneumonia and relatively high one-year mortality rates [1,2]. Moreover, the long-term immobility and bed stay predispose this population to severe deconditioning before LTx, increase postoperative complications, and delay recovery after LTx [3,4].

The distribution of donor lungs in Taiwan is based on both accumulated waiting time and medical urgency (risk of death without a transplant). In addition, the latter criterion was given priority over the former. Waiting list patients already dependent on invasive or noninvasive mechanical ventilator support are defined as 'respiratory failure' and are placed in 'status I' waitlists, whom are given first priority to obtain donor lungs. Due to the severe organ shortage, the long waiting time worsens the clinical condition of waitlists, and because the medical urgency of waitlist patients is a preferred criterion for organ allocation, 10 of 11 (91%) LTx procedures performed at National Taiwan University Hospital since 2006 have been for status I waitlist patients. In order to stabilize the hemodynamics of these critically ill patients and provide adequate oxygenation during transplantation, venoarterial (VA) extracorporeal membrane oxygenation (ECMO) support was routinely instituted through the groin area instead of cardiopulmonary bypass (CPB). This report summarizes the short-term results of bilateral sequential lung transplantations (BSLTx) performed under intraoperative VA ECMO support in 10 consecutive patients with respiratory failure.

## Materials and methods

### Study design

This retrospective cohort study was approved by the Institutional Research Board and included all consecutive cases of cadaveric BSLTx performed for patients with respiratory failure at the National Taiwan University Hospital (July 2006 to July 2008).

### Recipient and donor selection

In general, donor and recipient selection was in accordance with internationally accepted criteria [1,5,6]. Lung donor criteria were categorized as ideal or extended donor at our LTx institute. The ideal lung donor is less than 55 years of age, a nonsmoker, with a clear chest radiograph (CxR), a clear bronchoscopy result, and a partial pressure of arterial oxygen (PaO_2_)/fraction of inspired oxygen (FiO_2_) ratio of 350 mmHg or more with 5 mmHg positive end-expiratory pressure (PEEP). Extended donors are donors with lungs that meet most of the criteria but also have one or more of the following characteristics: PaO_2_/FiO_2 _ratio less than 350 mmHg with 5 mmHg PEEP, age more than 55 years, cumulative smoking history of more than 20 pack-years, CxR with localized substantial infiltrates, or positive results from Gram staining of airway lavage fluids.

### Donor management

A low-potassium dextran solution (Perfadex^®^, Vitrolife AB, Goteborg, Sweden) was used to perfuse the donor lung. Due to the wide use of extended donors, size-reduction (simple volume reduction or anatomic lobectomy) surgery before implantation was performed if parts of the donor lung looked unhealthy.

### ECMO circuit and lung transplantation technique

The ECMO circuit consisted of a centrifugal pump, a hollow-fiber microporous membrane oxygenator, and percutaneous thin-wall cannula (Medtronic Inc, Anaheim, CA, USA), all of which were coated with a heparin-bound Carmeda Bioactive surface. Except for one patient receiving ECMO support preoperatively due to pulmonary crisis in the ICU and its continued use in the operating theater for intraoperative support [7], VA ECMO was routinely instituted from the groin area under general anesthesia in the operating theater before pneumonectomy of the native lung. The 800 mL ECMO priming solution contains 1600 U heparin, the tubing sets in our ECMO circuit were heparin-bound, and it was expected that the duration of ECMO support for LTx procedure would not exceed 12 hours, so an additional intravenous bolus of heparin for systemic heparinization was not administered during transplantation. When a small femoral artery was found after exploration of the femoral vessels and the distal leg perfusion was not adequate after arterial cannulation, a small additional tube connected by a Y-adapter was inserted to the distal leg to prevent distal leg ischemia [8].

After VA ECMO support was set up, BSLTx was carried out through a clam shell incision. The ECMO blood flow during transplant procedure was set between 2 to 3 L/min according to the patient's clinical hemodynamic status. After completion of LTx, attempts were made to wean the patient off the ECMO system. If there were signs of severe reperfusion lung edema or acute primary graft dysfunction that did not allow the transplanted lung to function well immediately after transplantation, the ECMO support was continued during the move from the operating theater to the ICU. In the event of extension of the duration of ECMO support from temporary (in operating theater) to prolonged use (in the ICU), low-dose heparin was administered to keep activated clotting time at 160 to 180 seconds in order to prevent ECMO-related hemolysis or thrombosis complications.

### Postoperative management of the recipient

Patients were kept intubated for at least five days to maintain excellent expansion of the donor lungs and stayed in the ICU until they could cough sputum effectively. The choice of antibiotics was based on the results of sputum culture from donor and recipient. All patients were treated with a triple immunosuppressive regimen that included a calcineurin inhibitor (cyclosporine or tacrolimus), an antimetabolite (azathioprine or mycophenolate mofetil), and corticosteroids.

### Evaluation of pulmonary function after transplantation

To evaluate the postoperative pulmonary function changes over time, forced vital capacity (FVC) and forced expiratory volume in one second (FEV_1_) were measured at baseline preoperatively, and one month, three months, six months, and 12 months postoperatively if the patients could physically tolerate the spirometry test.

### Statistical analysis

Demographic and clinical characteristics of the patients are expressed as the mean ± standard deviation or proportions. In the spirometry analysis, pulmonary function variables (FVC, percent of predicted FVC, FEV_1_, and percent of predicted FEV_1_) were measured for each patient at time 0 (baseline), time 1 (1^st ^month postoperatively), time 2 (3^rd ^month postoperatively), time 3 (6^th ^month postoperatively), and time 4 (12^th ^month postoperatively). We performed repeated-measured analysis of variance with 'time' as the repeated variable to compare the variables of spirometry between different time points and the level of significance, Bonferroni-corrected α was set at 0.016667 (α = 0.05/c_1 _^4^, taking one from the four different postoperative time points for comparison with the baseline time 0) in the *post hoc *F test. Furthermore, we applied Huynh-Feldt ε correction to the degrees of freedom of the F test for terms in the model that involved repeated measures [9,10]. The software used was Stata 10.1 (StataCorp, College Station, TX, USA). The *P *values less than 0.05 and the *post-hoc P *values less than Bonferroni-corrected α were considered as statistically significant. Survival, in months, was calculated from the time of transplantation until date of death or end of the follow-up period (28 February, 2009). Cumulative survival following lung transplantation was determined using the Kaplan-Meier method.

## Results

A total of 10 consecutive status I waitlist patients were enrolled in the study, with a minimum follow-up of eight months. The time on the waiting list prior to transplantation was a mean of 19 months overall and the mean duration of post-transplant follow-up was 16.4 months. Seven of them were female and the mean body mass index of all patients was 17.8 kg/m^2 ^before LTx. Six patients depended on invasive ventilation support preoperatively, and five of these had tracheostomies. The other four patients had depended on noninvasive positive pressure ventilation (NIPPV) to provide adequate gas exchange before transplantation (Table 1). Before LTx, the mean PaO_2_/FiO_2 _ratio and partial pressure of arterial carbon dioxide (PaCO_2_) were 138 ± 72 and 68 ± 9 mmHg in the six intubated patients, and 287 ± 58 and 54 ± 8 mmHg in the four patients with NIPPV support, respectively.

**Table 1:** Patient characteristics, demographics, diagnosis for transplantation, pre-operative characteristics, and donor operations prior to transplantation in 10 patients receiving bilateral sequential lung transplantation under ECMO support

No.	Diagnosis	BMI	Pre-op ventilator support		Donor operation prior to LTx	
			
		(kg/m^2^)	IMV (days)	NIPPV	Lobectomy	Volume reduction
**1**	SLE PH	19	15*		RUL+RML+LUL	**-**
**2**	COPD	16		¶	-	**+**
**3**	SABO	21	148*		-	**-**
**4**	LAM	19		¶	-	**-**
**5**	Bronchiectasis	18	204*		RLL	**-**
**6**	BO	20		¶	RLL	**-**
**7**	LAM	16	180*		-	**-**
**8**	Cystic fibrosis	17	5#		-	**-**
**9**	Bronchiectasis	18	25*		RUL+LUL	**-**
**10**	LAM	15		¶	-	**+**

BMI = body mass index; BO = bronchiolitis obliterans; COPD = chronic obstructive pulmonary disease; IMV = invasive mechanical ventilation; LAM = lymphangioleiomyomatosis; LTx = lung transplantation; NIPPV = noninvasive positive pressure ventilation; RUL = right upper lobe; RML = right middle lobe; RLL = right lower lobe; LUL = left upper lobe; SABO = *Sauropus androgynus *bronchiolitis obliterans; SLE PH = systemic lupus erythematosus with pulmonary hypertension.

* receiving tracheostomy for long-term ventilation support before transplantation

# receiving endotracheal intubation for ventilation support before transplantation

¶ dependent on NIPPV support longer than six months

Before explantation, 6 of the 10 donors were categorized as extended donors for multifarious reasons (Table 2). Before implantation, four of them required lobectomies while the other two needed volume-reduction surgery (Table 1). The mean ischemic time for the first and second implanted lungs were 197 ± 53 and 330 ± 68 minutes. Eight of our ten patients were weaned off ECMO immediately after LTx and their mean duration of ECMO support was 7.8 ± 2.1 hours. Two patients could not be weaned off ECMO immediately post-transplantation (see next section) but were later smoothly weaned off on postoperative days 1 and 9 after lung graft recovery (Table 2). The mean length of ICU stay postoperatively was 43 days and the mean duration of in-hospital stay postoperatively was 70 days.

**Table 2:** Donor characteristics, pre-implantation donor management, donor ischemic time, and duration of weaning off ECMO support

Variable	Number (%) or mean ± SD
**Donor characteristics**	
Ideal donor	4 (40%)
	
Extended donor	6 (60%)
Reasons for extended donor classification	
Smoking history ≥; 20 pack-years*	3
PaO_2_/FiO_2 _ratio < 350 prior to explantation	2
Presence of purulent secretions	4
Infiltrate on chest x-ray	5
Positive sputum culture results from Gram stain	4
	
**Allograft ischemic time (minutes)**	
First implanted donor lung	197 ± 53
Second implanted donor lung	330 ± 68
	
**Duration of weaning off ECMO Support (n** ***= *** **10)**	
Weaned off ECMO support in OR (n = 8)	
Immediately after LTx (hours)	7.8 ± 2.1
Weaned off ECMO support in ICU (n = 2)	
Within 1 day after LTx	1 (10%)
> 1 day after LTx	1 (10%)

* One pack-year is defined as one pack of cigarettes smoked per day for one year.

ECMO = extracorporeal membrane oxygenation; FiO_2 _= fraction of inspired oxygen; LTx = lung transplantation; OR = operating room; PaO_2 _= partial pressure of arterial oxygen; SD = standard deviation.

### Postoperative complications

A total of four postoperative complications developed in our 10 LTx procedures. One patient needed re-exploration for right middle lobe (RML) lobectomy due to RML bronchus torsion after LTx. Two patients could not be weaned off ECMO in the operating theater due to severe reperfusion lung edema, which was strongly suspected to be a consequence of the use of extended donor organs with poor organ quality and the prolonged ischemic time resulting from lobectomies of donor lungs prior to implantation. One patient had a complicated postoperative course with localized impaired anastomotic healing, which healed gradually three weeks later without additional surgical intervention.

### Pulmonary functional test and outcome

**Figure 1:**
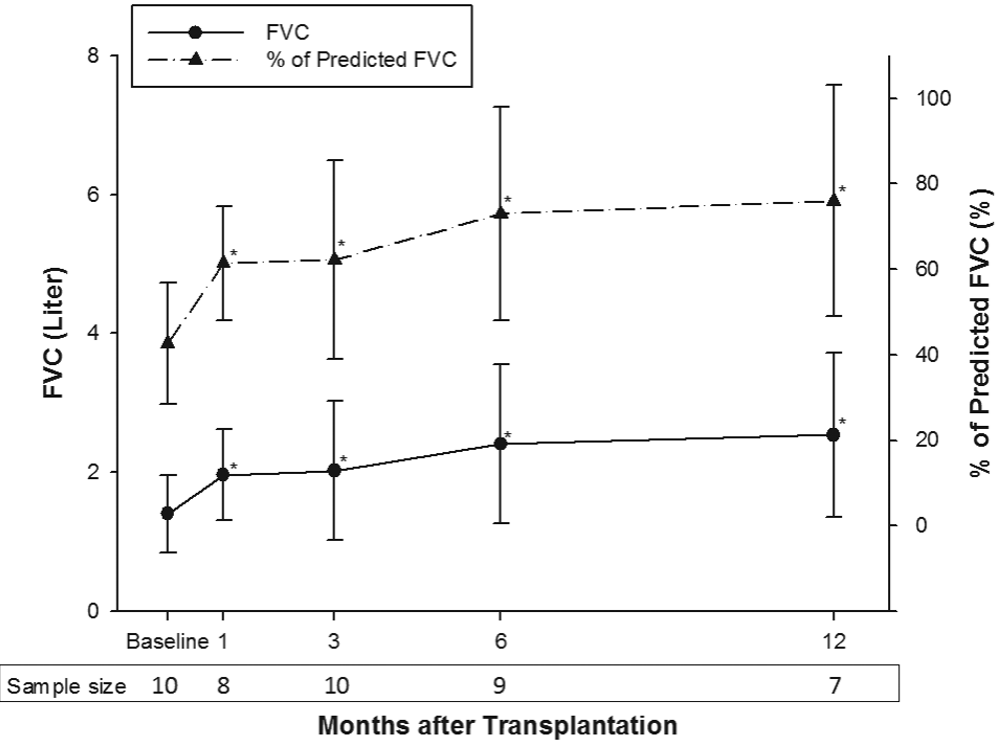
The mean values of forced vital capacity before and after transplantation. The mean forced vital capacity (FVC) increased significantly from 1.41 ± 0.56 L observed at baseline (43 ± 14% of predicted, n = 10) to 1.97 ± 0.66 L at 1 month (61 ± 13%; n = 8), 2.02 ± 1.00 L at 3 months (62 ± 23%; n = 10), 2.41 ± 1.15 L at 6 months (73 ± 25%; n = 9), and 2.54 ± 1.18 L at 12 months (76 ± 27%; n = 7) postoperatively. Values in the lower box indicate the number of patients undergoing spirometry tests at each time point. Solid lines represent the values of FVC at baseline and at 1, 3, 6, and 12 months after transplantation. Dashed lines represent estimated values of the FVC (percent of predicted). Significant differences: * *P *< 0.05 versus the baseline measurements.

**Figure 2:**
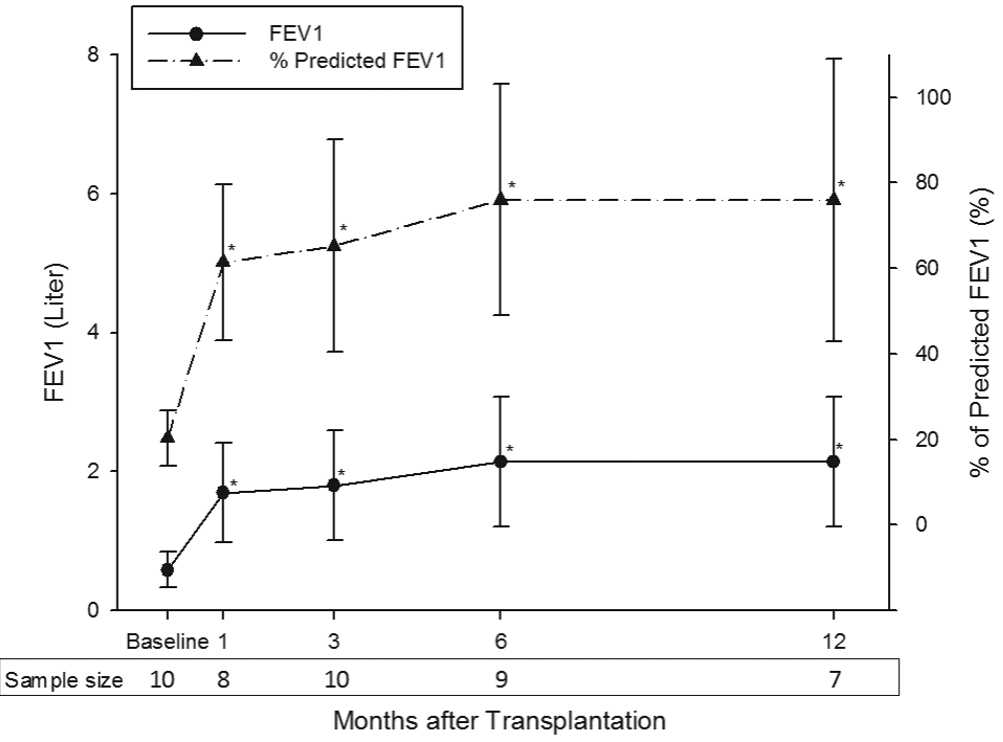
The mean values of forced expiratory volume in one second before and after transplantation. The mean forced expiratory volume in one second (FEV_1_) also increased significantly from 0.59 ± 0.26 L observed at baseline (20 ± 6% predicted; n = 10) to 1.69 ± 0.72 L at 1 month (61 ± 18%; n = 8), 1.8 ± 0.79 L at 3 months (65 ± 25%; n = 10), 2.14 ± 0.93 L at 6 months (76 ± 27%; n = 9), and 2.14 ± 0.94 L at 12 months (76 ± 33%; n = 7) postoperatively. Values in the lower box indicate the number of patients undergoing spirometry tests at each time point. Solid lines represent the values of FEV_1 _at baseline and at 1, 3, 6, and 12 months after transplantation. Dashed lines represent the estimated values of the FEV_1 _(percent of predicted). Significant differences: * *P *< 0.05 versus the baseline measurements.

By 28 February, 2009, 10 patients had received BSLTx longer than 3 months, 9 patients longer than 6 months, and 7 patients longer than 12 months. At the first month postoperatively, two patients suffered from postoperative complications and were too weak to perform pulmonary functional tests. The mean FVC and percent of predicted FVC rose sharply in the first month after LTx, then steadily improved in the first one year (Figure 1). A similar improvement trend was also observed in FEV_1 _and percent of predicted FEV_1 _(Figure 2).

There were two mortalities during the observation period. One patient died at five months due to sepsis resulting from profound pneumonia, while another died from chronic rejection at 19 months. By February 2009, 8 of the 10 patients were still alive and the cumulative survival rates at 3 months and at 12 months post-transplantation were 100% and 90%, respectively.

## Discussion

In this report, we describe the experience of 10 ventilator-dependent patients who underwent BSLTx via intraoperative VA ECMO support. There was neither postoperative nor in-hospital mortality and the pulmonary function values showed significant and continued improvement during the postoperative 12 months. Although BSLTx in this critical population had varied surgical complications and they needed longer ICU and hospital stays postoperatively, all the patients observed in this study were able to tolerate the transplant procedures. The 3-month and 12-month post-transplantation survival rates were 100% and 90%, respectively.

Since May 2005, a new allocation system was implemented in the United States that allocates donor lungs on the basis of medical urgency (risk of dying without transplant) and the net transplant benefit (opportunity for post-transplant survival) to avoid performing futile transplants [11]. In Taiwan, the total number of LTx was less than 120 in the period to February 2009 and it was very difficult to identify the factors associated with post-transplant survival in this small cohort of patients with diverse diagnoses. As the net transplant benefits are not calculated, this system can not avoid preferentially allocating scarce donor lungs to severely ill patients. Without any doubt, however, the allocation policy that top priority should be given to patients with the least amount of time to live and the current phenomenon in Taiwan whereby large numbers of LTx are performed in critically ill individuals indicate that this allocation system is not perfect and needs further detailed revision in the future.

Although outcomes of LTx have improved substantially in the past decade, the hospital mortality is still significant (10 to 15%) and the actuarial survival rates are 88% at three months and 81% at one year [12,13]. The high degree of illness of preoperative waitlisted patients was recognized as one of the major reasons contributing to the complicated postoperative recovery and high in-hospital mortality rate. Therefore, few LTx procedures were performed for ventilator-dependent recipients. Meyers and colleagues reported 21 of their 500 LTx procedures (4.2%) were performed for preoperative ventilator-dependent recipients during an observation period of 12 years [14]. Half of them required CPB support during the transplant and three hospital deaths (14%) occurred. Baz and colleagues reported their results of nine LTx procedures for ventilator-dependent patients who were ambulatory and able to undergo exercise therapy prior to LTx in their study period of five years at two well-known centers [15]. The one-year survival rate was 78% and the author emphasized that their recipients were selected from medically stable patients, not including more critically or acutely ill recipients. Contrary to their selected study individuals, all of our 10 consecutive recipients enrolled in the two-year study period were almost completely bed-ridden without being able to exercise before LTx. Although our in-hospital mortality rate and one-year survival rate were better than in the reports by Meyer and colleagues and Baz and colleagues, the long-term survival status still needs further observation.

The feasibility, benefits and complications of replacing CPB with ECMO in LTx operations have been well documented [7,16-19]. A German group reported their two-year experience of eight patients receiving LTx under ECMO support with an increased 90-day mortality rate (37.5%) due to infectious complications [18]. They discussed the advantages of femoral cannulation of ECMO circuits rather than conventional central connections of CPB in LTx procedures, which led to an undisturbed operative field. The Vienna group reported their large ECMO experience for intraoperative hemodynamic support in 147 LTx patients with excellent three-month (85.4%), one-year (74.2%), and three-year (67.6%) survival rates [19]. However, 33 of their 147 patients (22%) developed postoperative bleeding complications. Two patients developed major complications of cerebral bleeding intraoperatively and 31 patients needed postoperatively surgical revision due to bleeding problems. Although using the heparin-bound tubing sets, the Vienna group routinely administered an additional intravenous bolus of 75 IU/kg heparin before ECMO cannulation and they suspected that the level of systemic heparinization was too low to cause these bleeding complications. In contrast to their policy of giving an extra bolus of heparin for systemic heparinization, we did not add systemic heparin during the ECMO cannulation and intraoperative period.

Based on our previous ECMO life-support experience, we believe that the intraoperative complications of symptomatic thrombosis due to lack of systemic heparinization in the heparin-bound ECMO circuits with short duration usage (within 12 hours) was very low. In our cases, there was actually no sign of systemic or localized thrombosis developing during the LTx operation. Furthermore, none of our patients needed re-exploration due to postoperative bleeding from the thoracic cavity. Due to the relatively small number in our group, whether or not an additionally intravenous bolus of heparin into the ECMO circuits would be a primary contributor to intraoperative and post-transplant bleeding complications still needs further investigation. However, we believe that the short-term use of heparin-bound ECMO circuits without additional systemic heparinization will minimize coagulation disturbances and could effectively reduce postoperative bleeding complications during LTx.

## Conclusions

Respiratory failure patients depended on chronically ventilator support could tolerate the LTx procedures well with intraoperative ECMO assistance. Although varying degrees of postoperative complications and longer ICU and hospital stays delayed the post-transplant recoveries, the adequate level of regained pulmonary function and the satisfactory postoperative short-term survival suggest that LTx in these critically ill recipients still remains technically feasible, safe, and clinically meaningful.

## Key messages

• Performing LTx in respiratory failure patients had varying degrees of postoperative complications and longer ICU and hospital stays.

• Intraoperative ECMO assistance could provide adequate hemodynamic support in this critical population during the lung transplant procedure.

• Avoiding additional intravenous bolus of heparin into the ECMO circuits could minimize coagulation disturbances during LTx and effectively reduce postoperative bleeding complications.

• The adequate level of regained pulmonary function and the satisfactory postoperative short-term survival suggest that LTx in these critically ill recipients still remains technically feasible, safe, and clinically meaningful.

## Abbreviations

BSLTx: bilateral sequential lung transplantation; CPB: cardiopulmonary bypass; CxR: chest x-ray; ECMO: extracorporeal membrane oxygenation; FEV_1_: forced expiratory volume in one second; FiO_2_: fraction of inspired oxygen; FVC: forced vital capacity; LTx: lung transplantation; NIPPV: noninvasive positive pressure ventilation; PEEP: positive end-expiratory pressure; PaCO_2_: partial pressure of arterial carbon dioxide; PaO_2_: partial pressure of arterial oxygen; RML: right middle lobe; VA: venoarterious.

## Competing interests

The authors declare that they have no competing interests.

## Authors' contributions

HHH, JSC, SCH, SWK, PMH, NHC, CCC, and YCL were all involved in the transplant surgery, including the donor operation and recipient transplantation. SCH and WJK set up and maintained the ECMO life support system. RJC made substantial contributions to analysis and interpretation of data. HHH has been involved in drafting the manuscript and also made substantial contributions to conception and design of the study, and acquisition of data. YCL was involved in the conception of the study, revising the draft critically for important intellectual content and gave final approval of the version to be published. HHH, JSC, and SWK were also involved in the postoperative patients' care. All authors read and approved the final manuscript.
